# Potential Exergy Storage Capacity of Salt Caverns in the Cheshire Basin Using Adiabatic Compressed Air Energy Storage

**DOI:** 10.3390/e21111065

**Published:** 2019-10-30

**Authors:** Mark Dooner, Jihong Wang

**Affiliations:** School of Engineering, The University of Warwick, Coventry CV4 7AL, UK; jihong.wang@warwick.ac.uk

**Keywords:** compressed air energy storage, exergy, energy storage, exergy destruction, energy analysis, salt caverns

## Abstract

As the number of renewable energy sources connected to the grid has increased, the need to address the intermittency of these sources becomes essential. One solution to this problem is to install energy storage technologies on the grid to provide a buffer between supply and demand. One such energy storage technology is Compressed Air Energy Storage (CAES), which is suited to large-scale, long-term energy storage. Large scale CAES requires underground storage caverns, such as the salt caverns situated in the Cheshire Basin, UK. This study uses cavern data from the Cheshire Basin as a basis for performing an energy and exergy analysis of 10 simulated CAES systems to determine the exergy storage potential of the caverns in the Cheshire Basin and the associated work and power input and output. The analysis revealed that a full charge of all 10 caverns could store 25.32 GWh of exergy, which can be converted to 23.19 GWh of work, which requires 43.27 GWh of work to produce, giving a round trip efficiency of around 54%. This corresponds to an input power of 670.07 GW and an output power of 402.74 GW. The Cheshire Basin could support around 100 such CAES plants, giving a potential total exergy storage capacity of 2.53 TWh and a power output of 40 TW. This is a significant amount of storage which could be used to support the UK grid. The total exergy destroyed during a full charge, store, and discharge cycle for each cavern ranged from 299.02 MWh to 1600.00 MWh.

## 1. Introduction

To address climate change and limited fossil fuel resources, renewable energy technologies such as solar panels and wind turbines are increasingly being installed onto power grids. It is likely that renewable sources will become the dominant source of power in the near future. However, increasing the amount of renewable energy sources connected to the grid causes challenges that must be met. Namely, renewable energy sources tend to be intermittent in nature, and thus, it is difficult to match supply with demand. Energy Storage (ES) technologies present a solution to this challenge by allowing the generation and consumption of power to be decoupled. Compressed Air Energy Storage (CAES) is one such ES technology.

The operation of a typical CAES system is as follows. Surplus electrical energy from the grid is used to drive a motor which in turn powers a compressor, or compressor chain, which compress air from ambient pressure to high pressures. After compression, the air must be cooled using heat exchangers. The high pressure air is stored in storage vessels, generally large underground caverns but smaller, aboveground vessels can be used. When electrical energy is required by the grid, the high pressure stored air is used to drive turbines, which in turn drive generators. Air is generally heated before expansion. There are multiple types of CAES system. The first is “conventional” CAES, or Diabatic-CAES (D-CAES), in which the heat energy after compression is discarded and air is heated prior to expansion by burning gas. Adiabatic-CAES (A-CAES) stores the heat energy from compression and uses this energy to heat the air prior to expansion, making it more efficient than D-CAES [1,2]. Isobaric CAES is a type of D-CAES or A-CAES in which the pressure of the storage vessel is kept constant [3]. Isothermal-CAES (I-CAES) is a CAES system in which the temperature variations of the compression and expansion processes are prevented, giving the highest possible efficiency of the CAES cycle [4,5]. Other types of CAES include Super Critical-CAES (SC-CAES) [6,7] and Underwater-CAES (U-CAES) [8,9]. There are currently two large-scale CAES plants in operation: Huntorf in Germany and McIntosh in Alabama, USA. Huntorf was commissioned in 1978 and has a rated generation capacity of 290 MW, with a round trip efficiency of 42%. McIntosh began operation in 1991 and has a generation capacity of 110 MW, with an efficiency of 54%. Both of these plants are D-CAES, with other types of CAES generally being at the demonstration plant and simulation stage of maturity [10].

Significant work has been done by multiple authors to define the characteristics and relative strengths of the available and potential ES technologies [10,11,12,13,14,15,16,17,18,19]. To summarise, the distinguishing features of CAES are its very high power rating (5 to 400 MW) and rated energy capacity (580 to 2860 MWh) and its low self discharge leading to a long storage duration (hours to months) and long lifetime (20 to 60 years). Negative characteristics of CAES include low power (0.5 to 2 W/L) and energy (2 to 6 Wh/L) densities, meaning that CAES plants generally need to be very large. They also have low Round Trip Efficiency (RTE) (currently around 50%). Another advantage of CAES is its low cost per unit power rating (400 to 1500 $/KW) and per unit storage capacity (2 to 140 $/kWh) [20], making it amongst the cheapest forms of ES despite its high initial capital cost. The characteristics of CAES mean that it is well suited for energy management grid services such as energy arbitrage, peak shaving, and time shifting.

Due to the large and expensive nature of CAES systems, much of the current research is based on simulation studies of CAES systems. A brief review of such studies is presented here.

An accurate simulation model of air storage caverns was developed in Reference [21] based on the Huntorf plant-using energy and mass balance equations. It was found that heat transfer from the air to the surrounding rocks has a significant effect on the behaviour of the cavern and, thus, should be included in a model.

Combined energy and exergy analyses of CAES plants to assess their performance have been widely applied in the literature. In Reference [22], the characteristics of conventional (or D-CAES), A-CAES, isobaric, and poly-generation CAES systems were analysed using an exergy analysis method. Each system was designed to deliver 1 kWh of exergy at the output, and the required input exergy and various losses were calculated. The work demonstrated that A-CAES produces less waste than D-CAES due to heat energy being stored; that isobaric CAES removes the need for a throttle valve prior to expansion and thus removes a loss from the system; and that poly-generation CAES can be suitable for distributed power systems that require electrical, heating, and cooling energy. More detailed studies were carried out for A-CAES systems in References [23,24], with more focus on the exergy destruction of each component. It was found in Reference [23] that the compressors and turbines caused the largest exergy destruction, along with certain control valves. An RTE of 50% was reported. The same components destroyed the most exergy in Reference [24], with an RTE of around 60% reported, depending on the system structure and the component parameters. A full system model of an A-CAES plant was developed in Reference [25], and an investigation into how the system efficiency is determined by the component parameters was carried out. Key parameters influencing system efficiency were found to be the isentropic efficiencies of the compressors and turbines and the effectiveness of the heat exchangers. The structure of the system also influenced the efficiency. The authors in Reference [26] specifically studied the effect of the heat exchangers on system efficiency and drew similar conclusions to Reference [25].

Combining A-CAES systems with Packed Bed Thermal Energy Storage (PBTES), in which the heat exchangers and Thermal Energy Storage (TES) are combined, has been the subject of recent research. In Reference [27], it was found that using PBTES could improve the efficiency of an A-CAES system by around 5%. An A-CAES system using PBTES was modelled in Reference [28], in which efficiencies in excess of 70% were suggested to be achievable. In Reference [29], the cycle performance of an A-CAES system with PBTES was studied using simulation, resulting in efficiencies of 56.5% when phase change material was used in the packed beds against 53.2% for rock filled beds (i.e., sensible heat storage).

A large source of exergy loss in CAES systems is the throttle valve used prior to expansion to ensure that the turbines operate at or close to their rated values. In isobaric CAES, the pressure of the air storage vessel is kept constant, generally by pumping liquid into and out of the air storage vessel to modify its volume, and thus, the throttle is not required. Studies such as References [3,30,31] show an improvement in performance of isobaric CAES over A-CAES (with References [30,31] reporting RTE improvements of around 5%) at the expense of system and control complexity.

Further energy and exergy analyses of CAES systems focus on improved modelling, such as Reference [32], which focuses on modelling the off-design performance of the turbines, or on analysing advanced CAES systems, such as Reference [33], which focuses on underwater CAES, and Reference [34], which focuses on combining CAES with other elements of a power system.

The final consideration when analysing and/or designing CAES systems is the storage vessel used; underground storage caverns are considered here. The types of underground facilities considered for CAES are salt caverns, porous rock, abandoned mines, and hard rock caverns. Of these, salt caverns are the cheapest and most flexible, in addition to having been used in both the Huntorf and McIntosh plants, and are thus considered to be the most promising structure [35].

The British Geological Survey (BGS) investigates the potential in the UK for salt caverns that can be used for CAES. A study released in 2018 [35] estimated the available salt cavern volume of the Cheshire Basin, UK suitable for CAES. It was estimated that just 1% of the available salt caverns could support up to 100 CAES storage facilities, each with roughly 16 caverns, with each cavern being over 100 m in height. In this paper, the cavern data collected by BGS in Reference [35] is used to model such a CAES storage facility to estimate the total exergy storage potential of the cavern, the work energy needed to compress the air, and the work energy recovered during expansion.

This novelty of this paper is the application of thermodynamic and exergy analyses of A-CAES systems combined with real cavern data from the Cheshire Basin, UK. Other studies in this area apply such analysis techniques to assumed cavern (or other storage vessel) parameters or use parameters from existing CAES caverns such as the Huntorf cavern. This paper also calculates the exergy storage capacity of the caverns, which is usually omitted from similar studies. Hence, the exergy storage capacity of the Cheshire Basin can be estimated, along with the associated input and output work of the A-CAES facilities. This gives a useful indication of the storage capacity that CAES can provide for the UK and other countries with similar salt cavern resources. The remainder of this paper is structured as follows: Firstly, the nature of the salt caverns and the design of the CAES system to be modelled are explained. Secondly, the equations used for the energy and exergy analyses of the CAES systems are given. Thirdly, the results of the study are presented, showing the total exergy stored at full charge, the work energy consumed or produced by each component, and the exergy destroyed by each component. A comparison of two CAES systems is also given, with the dynamics of the air and thermal storage units included. Finally, the results are discussed and conclusions are drawn.

## 2. Salt Cavern and CAES System Description

### 2.1. Salt Caverns

Salt cavern data from the study carried out by BGS [35] is used in this paper. BGS used Esri’s ArcGIS^®^ Graphical Information System (GIS) software to estimate potential cavern locations and sizes within the Cheshire Basin, UK. To ensure that the identified caverns were suitable for CAES and practical, a number of size, structure, and location constraints were applied to the caverns, as well as constraints forcing the caverns to avoid existing infrastructure. The caverns were assumed to be cylinders with a radius of 50 m, with a “shaping factor” added to account for rough walls, reducing the radius to 41.83 m. Furthermore, it was assumed that 25% of the material in a potential salt cavern would be insoluble, thus reducing the storage volume. With these constraints and for a depth range of 250 to 1300 m, 3880 caverns were estimated with a total volume of 1830 million cubic metres. For this study, a random subset of 10 caverns of which the heights were above 100 m (based on the Huntorf cavern) were used from the original 3880. A CAES facility of this size is comparable to existing underground gas storage currently in use in the Cheshire Basin.

Table 1 shows the parameters of the ten caverns analysed in this study. The initial air pressure of the cavern is considered to be the minimum pressure of the cavern. In addition to Table 1, all ten caverns have the following common parameter values: Initial air temperature =323.15 K, heat transfer coefficient =30 WK^−1^m^−2^, radius =41.83 m, surrounding rock density =2200 kgm^−3^, surrounding rock heat capacity =840 Jkg^−1^K^−1^, and the surrounding rock heat conductivity =5.2 Wm^−1^K^−1^.

### 2.2. A-CAES System Structure

**Figure 1:**
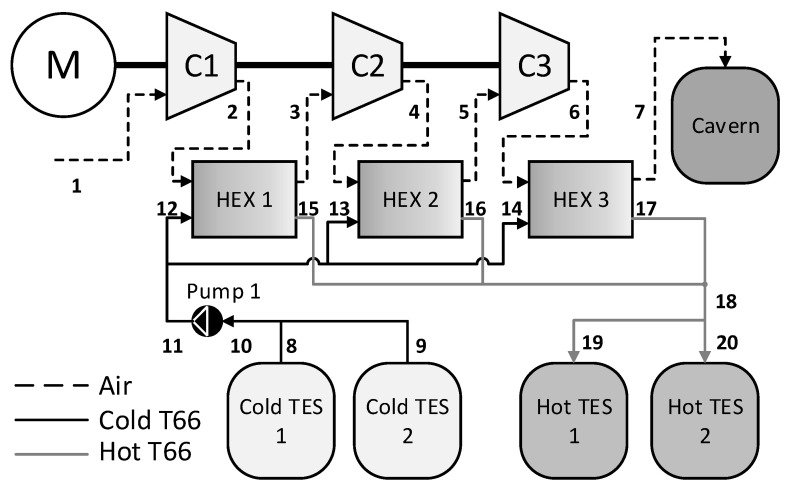
Adiabatic-Compressed Air Energy Storage (A-CAES) system charge design.

The structure of each of the 10 CAES systems is the same. The system is an A-CAES plant, in which the heat generated through the compression processes is stored and used to heat the air prior to expansion. The structure of the charging system is shown in Figure 1. The charge system is comprised of three compression layers, with each layer of a given system having the same compression ratio. Air leaving a compressor is directed through a heat exchanger, where it is cooled using thermal fluid. The heated thermal fluid is then stored in a TES unit. There are two cold TES units and two hot TES units, and a single pump moves the fluid from the cold units to the hot. All three compressors are on the same shaft, driven by a motor.

**Figure 2:**
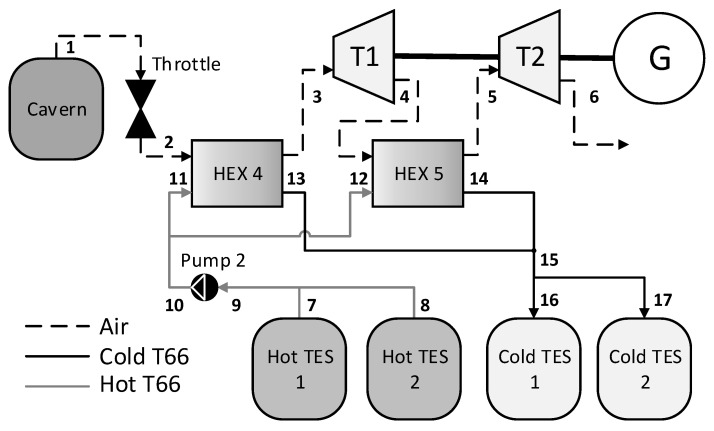
A-CAES system discharge design.

Figure 2 shows the structure of the discharge system. The air in the cavern flows through a throttle valve, of which the output pressure is equal to the minimum pressure of the cavern. There are two discharge layers, each with the same pressure ratio for a given system. Prior to expansion, the air is warmed via a heat exchanger using the hot thermal fluid stored after the charge phase. The cooled thermal fluid is then stored to be used in the next charge phase. Figure 1 and Figure 2 show the component names, along with the thermodynamic state numbers throughout the system. The storage units (i.e., the cavern and TES units) are common to each phase.

The thermal fluid used to cool and heat the air in the charge and discharge phases, respectively, and stored in the TES units is Therminol 66 (T66).

### 2.3. Simulation Study

The system shown in Figure 1 and Figure 2 is simulated for each cavern. Firstly, the caverns are charged to their maximum pressure with a constant air mass flow rate of 108 kg/s. Secondly, a storage period of 10 h is simulated, during which no air flows to or from the cavern. Finally, the caverns are discharged to their minimum pressure at a constant air mass flow rate of 108 kg/s.

At each state shown in Figure 1 and Figure 2, the pressure, temperature, enthalpy, and mass flow rate is calculated and the dynamics of the storage components is solved. The work done by or consumed by each component is also calculated. The total exergy stored at full charge and the exergy destruction of each component can then be determined.

## 3. Mathematical Model

This section gives the equations to be solved that model the behaviour of the A-CAES systems. An energy analysis is performed to solve for the thermodynamic states of the system and the behaviour of the storage components. Then, an exergy analysis calculates the exergy destroyed by each component and the exergy stored by the cavern and TES units. The following assumptions are made to simplify the analysis:

1. The air acts as an ideal gas.
2. The system in any operation reaches steady state.
3. The kinetic and potential energy changes are negligible.
4. The isentropic efficiencies of compressors, pumps, and turbines are fixed. Their values are given in Table 2.
5. The throttling process is isenthalpic.
6. The heat and pressure loss in the pipes connecting the components is negligible.
7. The ambient pressure and temperature are constant and equal to 101.32 kPa and 293.15 K, respectively.

### 3.1. Energy Analysis

The energy analysis is concerned with the first law of thermodynamics and generally takes the form of an energy balance equation, such that

(1) $$\dot{Q}+\dot{W}=\sum {\dot{m}}_{\mathrm{out}}h_{\mathrm{out}}-\sum {\dot{m}}_{\mathrm{in}}h_{\mathrm{in}},$$

where $\dot{Q}$ is the heat transfer rate, $\dot{W}$ is the rate of work produced/consumed, $\dot{m}$ is the mass flow rate of the air or thermal fluid, and *h* is the specific enthalpy of the air or thermal fluid. Subscripts “in” and “out” describe the fluid flow into and out of a component, respectively.

The thermodynamic properties such as enthalpy, entropy, and specific heat capacity of the air and thermal fluid are calculated using the CoolProp software, available at http://www.coolprop.org (accessed on 30 October 2019).

#### 3.1.1. Compressor Model

The change in temperature of the air at the outlet of a compressor is given by

(2) $$T_{c,\mathrm{out}}=T_{c,\mathrm{in}}\left\{1+\left(\pi_{c}^{\frac{\gamma -1}{\gamma }}-1\right)/\eta_{c}\right\},$$

where $T_{c,\mathrm{out}}$ is the outlet temperature, $T_{c,\mathrm{in}}$ is the inlet temperature, $\pi_{c}$ is the pressure ratio, γ is the ratio of specific heats, and $\eta_{c}$ is the isentropic efficiency.

The work consumed by each compressor is

(3) $${\dot{W}}_{c}={\dot{m}}_{c}\left(h_{c,\mathrm{out}}-h_{c,\mathrm{in}}\right),$$

where ${\dot{W}}_{c}$ is the compressor work, ${\dot{m}}_{c}$ is the mass flow rate of the air through the compressor, $h_{c,in}$ is the input enthalpy of the air, and $h_{c,out}$ is the output enthalpy.

#### 3.1.2. Turbine Model

The temperature of the air decreases during expansion and is modelled by

(4) $$T_{t,\mathrm{out}}=T_{t,in}\left\{1-\eta_{t}\left(1-\pi_{t}^{\frac{\gamma -1}{\gamma }}\right)\right\},$$

where $T_{t,out}$ is the outlet temperature, $T_{t,in}$ is the inlet temperature, $\eta_{t}$ is the isentropic efficiency of the turbine, and $\pi_{t}$ is the pressure ratio. The work produced by each turbine is

(5) $${\dot{W}}_{t}={\dot{m}}_{t}\left(h_{t,in}-h_{t,out}\right),$$

where ${\dot{W}}_{t}$ is the produced work, ${\dot{m}}_{t}$ is the mass flow rate of the air through the turbine, $h_{t,in}$ is the input enthalpy, and $h_{t,out}$ is the output enthalpy.

#### 3.1.3. Pump Model

The pumps used to pump the thermal fluid around the system is modelled in the same way as the compressors; thus, the output temperature of the thermal fluid and the work consumed by the pump are given, respectively, by

(6) $$T_{p,\mathrm{out}}=T_{p,\mathrm{in}}\left\{1+\left(\pi_{p}^{\frac{\gamma -1}{\gamma }}-1\right)/\eta_{p}\right\},$$

and

(7) $${\dot{W}}_{p}={\dot{m}}_{p}\left(h_{p,\mathrm{out}}-h_{p,\mathrm{in}}\right),$$

where the symbols have the same meaning as the compressor equations of Equations (2) and (3), with subscript “p” denoting the “pump”.

#### 3.1.4. Heat Exchanger Model

The energy balance of the heat exchangers in the charging process (i.e., components that cool the air and warm the thermal fluid) is

(8) $${\dot{m}}_{\mathrm{air}}\left(h_{\mathrm{air},\mathrm{in}}-h_{\mathrm{air},\mathrm{out}}\right)={\dot{m}}_{\mathrm{fluid}}\left(h_{\mathrm{fluid},\mathrm{out}}-h_{\mathrm{fluid},\mathrm{in}}\right),$$

and of the heat exchangers used in the discharging process (i.e., components that heat the air and cool the thermal fluid) is

(9) $${\dot{m}}_{\mathrm{air}}\left(h_{\mathrm{air},\mathrm{out}}-h_{\mathrm{air},\mathrm{in}}\right)={\dot{m}}_{\mathrm{fluid}}\left(h_{\mathrm{fluid},\mathrm{in}}-h_{\mathrm{fluid},\mathrm{out}}\right),$$

where $\dot{m}$ is mass flow rate through the heat exchanger; *h* is the enthalpy; subscripts “air” and “fluid” distinguish between the air and fluid flows, respectively; and subscripts “in” and “out” distinguish between the inlets and outlets, respectively.

The effectivenesses of a heat exchanger is defined as the ratio of the actual rate of heat transfer to the maximum and can be expressed as

(10) $$\epsilon =\frac{{\left(\dot{m}c_{p}\Delta T\right)}_{\mathrm{hot}\;\mathrm{or}\;\mathrm{cold}}}{{\left(\dot{m}c_{p}\right)}_{\min}\left(T_{\mathrm{hot},\mathrm{in}}-T_{\mathrm{cold},\mathrm{in}}\right)},$$

where the effectiveness is given the symbol ϵ; ${\left(\dot{m}c_{p}\right)}_{\min}$ is the smaller heat capacity rate between the air and thermal fluid streams; and subscripts “hot” and “cold” distinguish between the hot and cold fluid flows, respectively, depending on the position of the heat exchanger in the CAES system. The output temperature of the fluids flowing through the heat exchangers and the mass flow rate of the thermal fluid are calculated using a combination of Equations (8)–(10).

#### 3.1.5. Throttle Model

The energy balance of the throttle is given by

(11) $$h_{\mathrm{thr},\mathrm{in}}=h_{\mathrm{thr},\mathrm{out}}.$$

The input pressure and temperature of the throttle will be determined by the current pressure and temperature inside the cavern, and the output pressure of the throttle valve is a parameter set to the minimum pressure of the cavern. Thus, Equation (11) is used primarily to calculate the output temperature of the air.

#### 3.1.6. Cavern Model

The salt cavern model is based on the equations derived in Reference [36] and verified using the Huntorf plant. The model approximates a cylindrical cavern, and the heat transfer between the air inside the cavern and the surrounding rock is modelled using a Convective Heat Transfer (CHT) method.

The rate of change of pressure in the cavern, ${\dot{P}}_{\mathrm{cav}}$, is described by Equation (12), and the rate of change of temperature, ${\dot{T}}_{\mathrm{cav}}$, is given by Equation (13).

(12) $${\dot{P}}_{\mathrm{cav}}=\frac{1}{V_{\mathrm{cav}}}\left(\gamma RT_{\mathrm{in}}{\dot{m}}_{\mathrm{in}}-\gamma RT_{\mathrm{cav}}{\dot{m}}_{\mathrm{out}}+\left(\gamma -1\right)\lambda_{W}A_{W}\left(T_{W}-T_{\mathrm{cav}}\right)\right),$$

(13) $${\dot{T}}_{\mathrm{cav}}=\frac{1}{m_{\mathrm{cav}}c_{p}}\left(\lambda_{W}A_{W}\left(T_{W}-T_{\mathrm{cav}}\right)+{\dot{m}}_{\mathrm{in}}c_{p}\left(T_{\mathrm{in}}-T_{\mathrm{cav}}\right)+V_{\mathrm{cav}}{\dot{P}}_{\mathrm{cav}}\right),$$

where $V_{\mathrm{cav}}$ is the volume of the cavern, *R* is the gas constant, $\lambda_{W}$ is the heat transfer coefficient between the air and the surrounding rock, $A_{W}$ is the surface area of the cavern wall, $T_{W}$ is the temperature of the surrounding wall, and $m_{\mathrm{cav}}$ is the mass of air inside the cavern.

The heat loss through the cavern walls is modelled using

(14) $$\rho_{s}c_{p,s}\frac{dT_{s}}{dt}=\frac{1}{r}\frac{\partial }{\partial r}\left(k_{s}r\frac{\partial T_{s}}{\partial r}\right),$$

with the following boundary conditions

(15) $$r=r_{W},\;-k_{s}\frac{\partial T_{s}}{\partial r}=\lambda_{W}\left(T_{W}-T\right),$$

(16) $$r=r_{\infty },\;T_{s}=T_{0},$$

where $\rho_{s}$ and $c_{p,s}$ are the density and heat capacity of the surrounding rock, respectively; $r_{W}$ is the radius of the cavern; $k_{s}$ is the heat conductivity of the surrounding rock; and $T_{s}$ is the temperature of the surrounding rock. Equations (14)–(16) are solved using the Method of Lines (MOL).

#### 3.1.7. Thermal Energy Storage Model

The thermal fluid storage vessels are assumed to be cylindrical and to undergo heat transfer through the walls to the surrounding air. The TES model consists of three heat flow rate equations describing the input heat rate due to inflowing thermal fluid (Equation (17)), the output heat rate due to outflowing fluid (Equation (18)), and the heat loss through the walls of the vessel (Equation (19)). These three equations are given as follows:

(17) $${\dot{Q}}_{\mathrm{in}}={\dot{m}}_{\mathrm{in}}c_{p}\left(T_{\mathrm{in}}-T_{\mathrm{TES}}\right),$$

(18) $${\dot{Q}}_{\mathrm{out}}={\dot{m}}_{\mathrm{out}}c_{p}\left(T_{\mathrm{out}}-T_{\mathrm{TES}}\right),$$

and

(19) $${\dot{Q}}_{\mathrm{loss}}=\frac{2\pi k_{\mathrm{TES}}\left(T_{0}-T_{\mathrm{TES}}\right)}{\ln\frac{r_{2}}{r_{1}}},$$

where $\dot{Q}$ is the heat transfer rate, $k_{\mathrm{TES}}$ is the thermal conductivity of the walls, $T_{\mathrm{TES}}$ is the temperature inside the TES vessel, $T_{0}$ is the temperature of the surrounding air; and $r_{1}$ and $r_{2}$ are the radii of the cavern to the inner and outer walls, respectively.

The change in the height, ${\dot{H}}_{\mathrm{TES}}$, of the fluid inside the TES vessel is given as

(20) $${\dot{H}}_{\mathrm{TES}}=\frac{\left({\dot{m}}_{\mathrm{in}}-{\dot{m}}_{\mathrm{out}}\right)}{\rho A_{\mathrm{TES}}},$$

where ρ is the density of the thermal fluid and $A_{\mathrm{TES}}$ is the cross-sectional area of the vessel. The change in temperature of the fluid inside the vessel, ${\dot{T}}_{\mathrm{TES}}$, is

(21) $${\dot{T}}_{\mathrm{TES}}=\frac{\left({\dot{Q}}_{\mathrm{in}}-{\dot{Q}}_{\mathrm{out}}\right)+{\dot{Q}}_{\mathrm{loss}}}{c_{p}\rho A_{\mathrm{TES}}H_{\mathrm{TES}}}.$$

Parameters for the components described in this section are given in Table 2.

### 3.2. Exergy Analysis

Exergy is defined as the portion of energy that can be used to perform useful work [37]. The total exergy in a flow stream consists of four components: physical, chemical, kinetic, and potential. In studying CAES systems (and assuming that there is no combustion), there is no chemical exergy component and the kinetic and potential exergy are neglected. Thus, only physical exergy is considered, of which the rate can be defined as

(22) $${\dot{E}}_{x}^{PH}=\dot{m}e_{x},$$

where $e_{x}$ is the specific exergy and is defined as

(23) $$e_{x}=\left(h-h_{0}\right)-T_{0}\left(s-s_{0}\right),$$

where *s* is the entropy of the fluid flow and $h_{0}$ and $s_{0}$ are the ambient enthalpy and entropy, respectively.

In this study, the concern is the destruction of the exergy by each component and the amount of exergy stored in the cavern and the TES units. The equations describing the exergy destruction rate by the compressors (Equation (24)), turbines (Equation (25)), pumps (Equation (26)), throttle (Equation (27)), and heat exchangers (Equation (28)) are given below:

(24) $${\dot{E}}_{x,c}^{d}={\dot{m}}_{\mathrm{in}}e_{x,\mathrm{in}}-{\dot{m}}_{\mathrm{out}}e_{x,\mathrm{out}}+{\dot{W}}_{c},$$

(25) $${\dot{E}}_{x,t}^{d}={\dot{m}}_{\mathrm{in}}e_{x,\mathrm{in}}-{\dot{m}}_{\mathrm{out}}e_{x,\mathrm{out}}-{\dot{W}}_{t},$$

(26) $${\dot{E}}_{x,p}^{d}={\dot{m}}_{\mathrm{in}}e_{x,\mathrm{in}}-{\dot{m}}_{\mathrm{out}}e_{x,\mathrm{out}}+{\dot{W}}_{p},$$

(27) $${\dot{E}}_{x,\mathrm{thr}}^{d}={\dot{m}}_{\mathrm{thr}}\left(e_{x,\mathrm{in}}-e_{x,\mathrm{out}}\right),$$

(28) $${\dot{E}}_{x,\mathrm{HEX}}^{d}={\dot{m}}_{\mathrm{in},\mathrm{air}}e_{x,\mathrm{in},\mathrm{air}}+{\dot{m}}_{\mathrm{in},\mathrm{fluid}}e_{x,\mathrm{in},\mathrm{fluid}}-{\dot{m}}_{\mathrm{out},\mathrm{air}}e_{x,\mathrm{out},\mathrm{air}}-{\dot{m}}_{\mathrm{out},\mathrm{fluid}}e_{x,\mathrm{out},\mathrm{fluid}}.$$

The exergy stored in the cavern [36] is calculated using

(29) $${\dot{E}}_{x,\mathrm{cav}}=\left({\dot{m}}_{\mathrm{in}}-{\dot{m}}_{\mathrm{out}}\right)\left\{c_{p}\left(T-T_{0}\right)-T_{0}\left(c_{p}\ln\frac{T}{T_{0}}-R\ln\frac{P}{P_{0}}\right)\right\},$$

and the exergy stored in the TES units is calculated using

(30) $${\dot{E}}_{x,\mathrm{TES}}=\left({\dot{m}}_{\mathrm{in}}-{\dot{m}}_{\mathrm{out}}\right)c_{p}\left\{\left(T_{\mathrm{TES}}-T_{0}\right)-T_{0}\ln\frac{T_{\mathrm{TES}}}{T_{0}}\right\},$$

where $P_{0}$ is the ambient pressure.

### 3.3. Performance Analysis

In this study, the RTE of the CAES system is used as a performance measure. The RTE is defined as the ratio between the discharge energy and the charge energy, i.e., the work produced by the turbines and the work consumed by the compressors and pumps, defined as

(31) $$\mathrm{RTE}=\frac{W_{\mathrm{expansion}}}{W_{\mathrm{compression}}}.$$

## 4. Results

This section is split into two parts: The first presents the total exergy stored, the work and RTE of each component, and the exergy destroyed by each set of components. Then, two systems (cavern 3 and cavern 7) are compared in detail, with the dynamics of the storage units and the thermodynamic states of the system presented, to highlight the effect of the cavern size on the system.

### 4.1. System Results

Table 3 shows the total final exergy stored inside the caverns for all 10 caverns, along with the combined totals. The initial exergy gives the exergy stored in the cavern when it is discharged, i.e., at its minimum pressure and a temperature of 323.15 K. The final exergy is the exergy stored in the caverns once they are fully charged, and the net exergy is the difference between the two. The net exergy represents the exergy that could actually be utilised because the caverns cannot be discharged below their minimum pressure.

Table 4 gives the work consumed by the compressors and pumps and the work produced by the turbines for each system. The total input and output work and the corresponding RTE are also provided.

Table 5 shows the exergy destruction of each component, with like components combined. The total exergy destruction of each system is also shown.

### 4.2. Case Study

In this section, the performance of two systems are compared. The two systems being compared are system/cavern 3 and system/cavern 7. These were chosen because cavern 3 has the largest volume and cavern 7 has the smallest. The thermodynamic states, exergy destruction by components, and dynamic performance of the storage elements are given.

Table 6 and Table 7 give the thermodynamic states of the charge phase for systems 3 and 7, respectively. The state numbers in the first column correspond to the numbers given in Figure 1. Note that the enthalpy of the air and T66 have different references for zero enthalpy. In the model, it is the difference between enthalpy values in between the inlet and outlet of components through which fluid flows that is used in calculations; thus, this does not introduce an error into the system.

Table 8 and Table 9 give the thermodynamic states of the discharge phase for systems 3 and 7, respectively. The state numbers in the first column correspond to the numbers given in Figure 2. Note that, throughout discharge, the temperature and pressure in the cavern and, thus, at the inlet to the throttle (state 1) are continuously changing. This causes the temperature of the air leaving the throttle valve to also continuously change, and thus, the thermodynamic states of the discharge system are different at each time step. For this reason, the thermodynamic states of the discharge system at the beginning and end of the discharging phase are given in Table 8 and Table 9.

**Figure 3:**
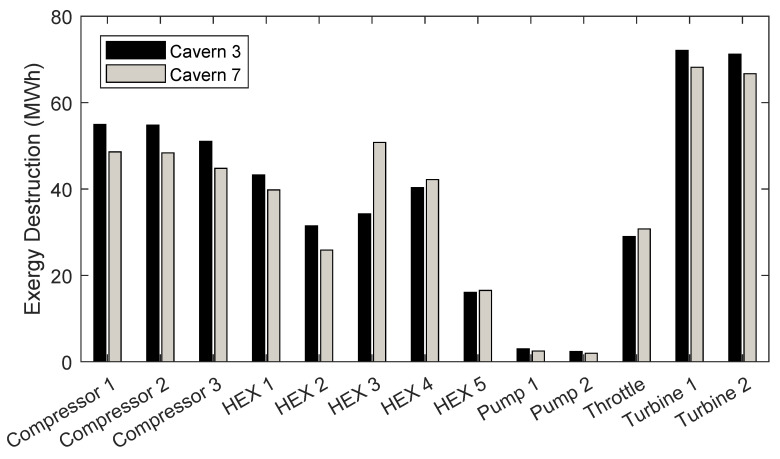
Component exergy destruction comparison.

Figure 3 compares the exergy destroyed by each component in systems 3 and 7.

**Figure 4:**
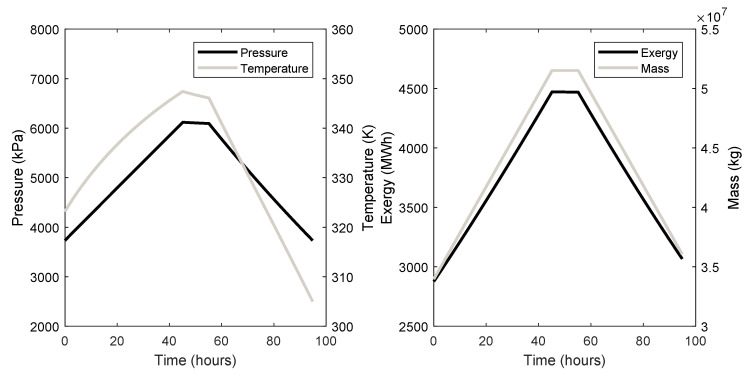
Cavern 3 dynamic behaviour.

**Figure 5:**
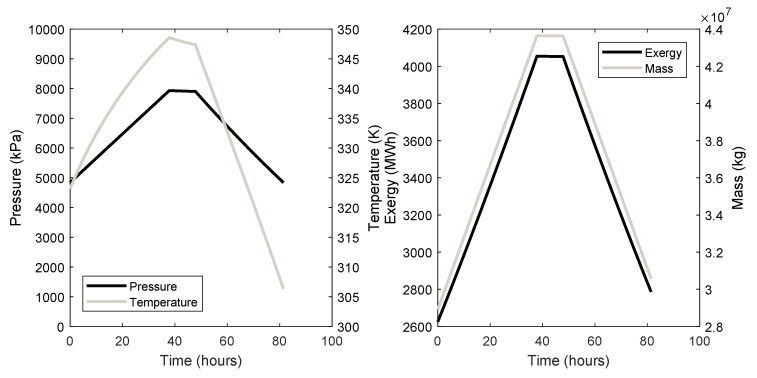
Cavern 7 dynamic behaviour.

The cavern dynamics, i.e., the pressure, temperature, exergy, and mass of the air inside the caverns through their charge, store, and discharge phases are given in Figure 4 for cavern 3 and Figure 5 for cavern 7.

**Figure 6:**
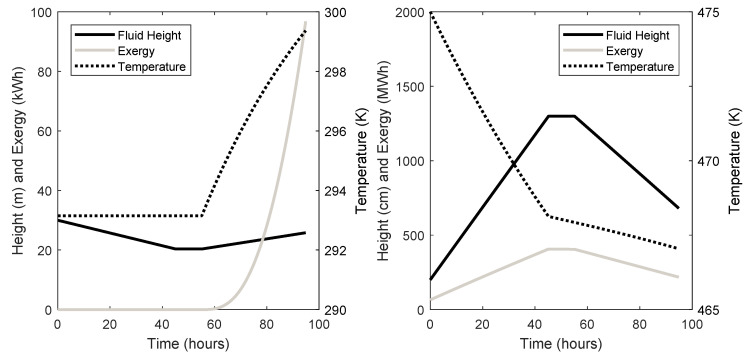
System 3 Thermal Energy Storage (TES) dynamics (left: cold store; right: hot store).

**Figure 7:**
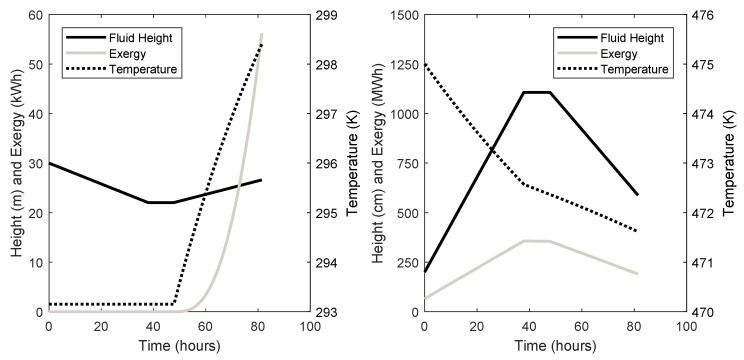
System 7 TES dynamics (left: cold store; right: hot store).

The dynamic behaviour of the cold and hot TES units are given in Figure 6 for system 3 and Figure 7 for system 7. Fluid height, temperature, and stored exergy are plotted throughout the charge, store, and discharge phases.

For system 3, the charge phase starts at 0 h and ends after around 45 h, after which the storage phase begins and lasts for 10 h. The discharge phase starts at around 55 h and ends at around 95 h. Likewise, for system 7, the charge phase starts at 0 h and ends after around 38 h, after which the storage phase begins and lasts for 10 h. The discharge phase starts at around 48 h and ends at around 81.5 h.

## 5. Discussion

### 5.1. Total Exergy Storage Capacity

The results shown Table 3 give the total exergy stored after a full charge for each of the 10 potential salt caverns in the Cheshire Basin of which the heights are equal to or greater than 100 m. The total exergy stored in the 10 caverns is 72,493.95 MWh, giving a net exergy of 25,326.30 MWh for each charge. The largest amount of net exergy stored in a single cavern was 3931.61 MWh in cavern 1, and the least was 995.63 MWh in cavern 4. Cavern 1 has the third highest maximum pressure but is a higher volume than the two caverns with higher maximum pressures (caverns 8 and 10). Likewise, cavern 4 is not the smallest cavern but has the lowest maximum pressure of the caverns analysed. These results demonstrate that this analysis, combined with suitably accurate cavern physical parameters, can identify which caverns should be utilised by maximise storage capacity. The results also demonstrate the large storage potential of CAES in the Cheshire Basin and other such environments. Assuming that the caverns analysed in this study are representative and noting that 100 such facilities are deemed possible in the Cheshire Basin [35], a potential total of around 2.5 TWh of exergy could be stored at full charge.

### 5.2. Work and Power

Table 4 shows the work consumed by the compressors and pumps during the discharge and (primarily) charge phases, along with the work produced by the turbines during the discharge phase. The total work produced is 23,190.80 MWh, which requires a work input of 43,273.68 MWh. This corresponds to a total input of power of 671.07 GW and a total output power of 402.74 GW for all 10 systems. Note that the output power is taken at the end of the discharge phase, when it is at its lowest. These values are heavily dependent on the efficiency of each component (which will vary with scale and operating conditions), the structure of the whole system, and the charge/discharge rates. However, these results give a good indication of the amount of power and work that can be supplied by such a CAES plant and the surplus energy generated by renewable sources that would be required to fully utilise the system. Assuming that these results are representative and that 100 such plants are developed [35], the Cheshire Basin could produce 2.3 TWh of work and deliver a total of 40 TW of power.

The RTE of systems range from 53.24% for cavern 6 and 54.94% for cavern 8. The values are similar because the predominant factors affecting the RTE are the isentropic efficiencies of the compressors, pumps, and turbines; the effectiveness of the heat exchangers; and the structure of the system. In this study, all of these values are constant and the same for each cavern. Values of isentropic efficiency and heat exchanger effectiveness will change with the operating conditions of their associated components and require models with higher fidelity than those used in this study to fully analyse.

### 5.3. Component Exergy Destruction

Table 5 shows that the system with the highest total exergy destruction over the whole charge/discharge cycle is system 1 at 1600.00 MWh. This is because, due to the cavern’s large volume, high maximum storage pressure, and high ambient rock temperature, the exergy storage capacity of this cavern is the highest out of the 10 caverns analysed. The exergy destruction breakdown given in Table 5 shows that, whereas the total amount of exergy destruction broadly scales with the total exergy stored, the ratio of exergy destruction for each set of components varies across each system despite the fact that the system design is the same. This implies that careful consideration and analysis during the design phase is required to minimise total exergy destruction and that no single component or set of components is generally responsible for exergy destruction in CAES systems.

For example, in system 1, the heat exchangers in the charge system destroy more exergy than the compressors, whereas in system 2, it is the other way round. Similarly, in system 1, the turbines destroy more exergy than the compressors, whereas in system 2, it is the compressors that destroy more exergy than the turbines. The amount of exergy destroyed by each component depends on the pressure and temperature changes across it, the mass flow rate of the fluid(s) through it, and the work that it consumes or produces (if any). These values are all highly dependent on factors such as the size of the cavern, the cavern’s maximum and minimum pressures, the pressure ratio of the compressors and turbines, the initial conditions of the heat stores, and the structure of the whole system. A CAES system such as those analysed here could be optimised to minimise exergy destruction.

### 5.4. Case Study

The performance of two systems designed for caverns 3 and 7 were compared to illustrate the thermodynamic states of the systems and the dynamic characteristics of the energy storage units, i.e., the caverns and TES. Cavern 3 is the largest cavern and 7 is the smallest in terms of volume, although not storage capacity, since this is also dependent on the maximum storage pressure. The thermodynamic states for the charge phase are given in Table 6 and Table 7, and the discharge phase thermodynamic states are given in Table 8 and Table 9. Analysis of these results demonstrates that the scale and design of the system directly affects the pressures, temperatures, and mass flow rates that the components are subject to. This is important information when designing a system.

The exergy destruction of each component, calculated using the thermodynamic states given in Table 6 to Table 9, are shown in Figure 3. The total exergy destruction of the two systems are close (503.60 MWh for system 3 and 486.86 MWh for system 7) owing to the similar exergy storage capacities of their caverns (1594.48 MWh for system 3 and 1429.25 MWh for system 7). Figure 3 demonstrates the sensitivity of component level exergy destruction to the system’s operating conditions. Despite destroying less exergy overall, the heat exchangers in system 7 destroy more exergy than the heat exchangers in system 3 (overall), even though they are operated for less time. This is due to the higher pressure ratio required for the compressors and expanders in system 7, meaning that the output temperatures of the compressors where higher, requiring the heat exchanger to pump more thermal fluid to cool the air before the next compression stage. Likewise, the air temperature after an expansion phase is lower for a turbine with a higher pressure ratio, and thus, more heating fluid was needed by the heat exchangers to heat the air before the next expansion phase. The turbines destroy the most exergy of each component. This is partly due to the fact that there are only two turbines used for expansion and three compressors for compression. Turbines also produce a large change in specific exergy because they affect both temperature and pressure, which results in large exergy destruction due to Equation (25). Reducing the exergy destruction of a particular component requires system level design and operation optimisation.

The cavern dynamics of both systems are shown in Figure 4 and Figure 5. Of importance are the timescales for a full charge to dischare cycle and how the temperature affects the exergy. Regarding the time taken to complete a whole cycle, both systems take around 2 days to fully charge and discharge. This value is dependent on the size and maximum pressure of the cavern and on the mass flow rate of the air. Clearly, these are long timescales and compressors and turbines with higher mass flow rates should be considered, which will require a higher power input. CAES will have inherently long charge times, and this must be taken into account when planning which grid services that it will provide. Considerations such as the amount of surplus energy required to charge the caverns and for how long the energy will be available should be made when planning CAES systems. It can be seen in Figure 4 and Figure 5 that the temperature of the air inside the caverns at the end of the discharge phase is lower than the beginning of the charge phase. This has the effect of reducing the amount of energy available during discharge. It can be seen that there is more exergy remaining in the cavern after discharge than at the start of the charge phase, representing a loss in the system.

Finally, the dynamics of the TES units can be seen in Figure 6 and Figure 7. It can be seen that the amount of fluid in the TES units and the temperature of the fluid after discharge is not equal to the states before the charge phase. There is also more exergy stored in each TES unit after discharge than before the charge phase, which could be seen as a loss in the system. This is important information when considering the design and control of the TES units. This will also have an effect on the cycle-to-cycle behaviour of the system, since the initial conditions of each phase are changeable.

### 5.5. Future Work

This study in primarily concerned with the exergy storage capacities and associated work input and output. Through this study, numerous recommendations for future work can be made.

The storage capacity of a cavern is determined by its size and its minimum and maximum storage pressures. Thus, the analysis presented in this work can be used to determine which caverns have the highest capacity and to represent the best investment for excavation. Combining this exergy and energy analysis with a cost analysis for dissolving the salt and construction, maintenance, and potential profit of the corresponding CAES plant would yield further useful results for planning new CAES facilities.

All of the systems analysed in this report have similar RTE of around 54%. This is because of the assumption that all component efficiencies across the system were the same and constant and that the structure of each system is the same. In reality, the component efficiencies will vary with scale and operating conditions and the structure of the system can be changed at the design phase. Improving the fidelity of the component models to incorporate more realistic efficiency behaviour and optimising the structure of the system would yield more accurate results and allow for more detailed design.

Fundamentally, any CAES system will be required to support grid operation. Thus, combining the analysis presented here with data regarding the availability of energy to charge the system and regarding the demand for energy supplied by the CAES system would give a more thorough analysis of the suitability of CAES for meeting future power grid requirements. Real supply and demand data would also mean that, rather than studying a full charge and discharge cycle, more realistic cycle behaviour and its effect on the performance of the CAES systems could be carried out.

## 6. Conclusions

In this study, the exergy storage capacity of 10 salt caverns situated in the Cheshire Basin, UK has been analysed. The associated work input and output, round trip efficiency, component level exergy destruction, thermodynamic states, and storage unit (air cavern and thermal energy storage units) dynamic behaviour of CAES plants incorporating the caverns have also been studied. The number and size of the analysed caverns were based on current gas storage facilities in in the Cheshire Basin and the Huntorf CAES plant in Germany.

The energy and exergy analyses revealed that a full charge of all 10 caverns could store a net exergy of 25.32 GWh, which can be converted to 23.19 GWh of work via the turbines, and requires 43.27 GWh of work to compress the air and pump the thermal fluid, giving a round trip efficiency of around 54%. This corresponds to an input power of 670.07 GW and an output power of 402.74 GW. It has been suggested that the Cheshire Basin could support around 100 such CAES plants [35], giving a potential total exergy storage capacity of 2.53 TWh and a power output of 40 TW.

The CAES systems with the smallest and largest caverns were studied in more detail. It was found that the scale of the system affects which components destroy the most amount of exergy, meaning that careful design and analysis are required in order to minimise exergy destruction. Also, the time taken to charge the caverns were 38 and 45 h for the smallest and largest caverns, respectively, at a constant mass flow rate of 108 kg/s. Higher mass flow rates or suitable design within a power system are required to mitigate such large charge times.

## Figures and Tables

**Table 1:** Cavern parameters.

Cavern ID	Initial Air Pressure (kPa)	Initial Air Mass (kg)	Cavern Volume (m^3^)	Cavern Surface Area (m^2^)	Maximum Pressure (kPa)	Ambient Rock Temp (K)	Height (m)
1	8667.65	7.09E + 07	7.58E + 05	47,251.08	14,209.27	307.47	138.01
2	3379.81	2.62E + 07	7.18E + 05	45,302.41	5540.68	292.12	130.59
3	3732.66	3.40E + 07	8.44E + 05	51,347.23	6119.12	294.21	153.60
4	2937.49	2.25E + 07	7.10E + 05	44,954.27	4815.55	290.91	129.26
5	8101.01	6.67E + 07	7.64E + 05	47,514.61	13,280.34	293.97	139.01
6	6789.32	6.09E + 07	8.32E + 05	50,778.41	11,130.03	291.36	151.43
7	4840.24	2.89E + 07	5.54E + 05	37,483.65	7934.82	295.37	100.83
8	8946.56	6.08E + 07	6.31E + 05	41,143.08	14,666.49	311.82	114.76
9	6943.81	4.21E + 07	5.62E + 05	37,855.57	11,383.30	301.81	102.24
10	9766.95	6.42E + 07	6.10E + 05	40,145.57	16,011.39	316.56	110.96

**Table 2:** Component parameters.

Parameter	Value	Unit
Compressor Isentropic Efficiency, $\eta_{c}$	0.9	N/A
Compressor Pressure Ratio, $\pi_{c}$	3.8–6.4	N/A
Turbine Isentropic Efficiency, $\eta_{t}$	0.9	N/A
Turbine Pressure Ratio, $\pi_{t}$	4.4–9.4	N/A
HEX Effectiveness, ϵ	0.95	N/A
HEX Pressure Drop	4%	N/A
Pump Isentropic Efficiency, $\eta_{p}$	0.7	N/A
Pump Pressure Ratio, $\pi_{p}$	1.8	N/A
Cold Store Initial Temp	293.15	K
Cold Store Initial Liquid Height	30	m
Cold Store Max Height, $H_{TES}$	30	m
Cold Store Area, $A_{TES}$	1500	m^2^
Cold Store Thermal Conductivity, $k_{TES}$	10	Wm^−1^K^−1^
Hot Store Initial Temp	475	K
Hot Store Initial Liquid Height	2	m
Hot Store Max Height, $H_{TES}$	30	m
Hot Store Area, $A_{TES}$	1500	m^2^
Hot Store Thermal Conductivity, $k_{TES}$	0.1	Wm^−1^K^−1^
Throttle Output Pressure	2937.49–9766.95	kPa

**Table 3:** Total exergy stored in fully charged caverns.

Cavern ID	Initial Exergy (MWh)	Final Exergy (MWh)	Net Exergy (MWh)
1	7400.80	11,332.41	3931.61
2	2154.44	3351.41	1196.97
3	2877.50	4471.98	1594.48
4	1779.68	2775.31	995.63
5	6861.64	10,543.50	3681.86
6	6012.71	9269.01	3256.30
7	2625.12	4054.37	1429.25
8	6397.11	9780.59	3383.49
9	4174.10	6409.30	2235.20
10	6884.55	10,506.05	3621.51
**Totals**	47,167.65	72,493.95	25,326.30

**Table 4:** Component work and Round Trip Efficiency (RTE).

Cavern ID	Comp 1 (MWh)	Comp 2 (MWh)	Comp 3 (MWh)	Pump 1 (MWh)	Pump 2 (MWh)	Turb 2 (MWh)	Turb 2 (MWh)	Total In (MWh)	Total Out (MWh)	RTE
1	2150.16	2272.66	2411.97	6.94	8.81	1893.03	1775.67	6850.53	3668.70	53.55%
2	627.01	653.06	675.81	2.82	3.21	535.74	520.88	1961.90	1056.62	53.86%
3	832.25	867.74	899.53	3.64	4.17	712.56	690.52	2607.32	1403.07	53.81%
4	516.8	537.12	553.96	2.45	2.77	433.03	433.03	1613.10	866.07	53.69%
5	1986.15	2095.46	2216.99	6.65	8.22	1736.74	1633.69	6313.47	3370.43	53.38%
6	1743.29	1834.04	1930.94	6.19	7.48	1509.38	1430.75	5521.95	2940.13	53.24%
7	757.49	793.03	827.81	3.00	3.55	652.81	627.17	2384.87	1279.97	54.67%
8	1837.09	1941.76	2060.78	5.93	7.47	1630.69	1526.25	5853.04	3156.94	54.94%
9	1208.14	1272.24	1341.69	4.20	5.15	1048.65	992.82	3831.43	2041.47	54.28%
10	1984.30	2101.16	2236.48	6.18	7.93	1643.9	1763.43	6336.06	3407.40	54.75%
**Totals**	13,642.66	14,368.29	15,155.96	48.00	58.76	11,796.59	11,394.21	43,273.68	23,190.80	N/A

**Table 5:** Exergy destruction.

Cavern ID	Compressors (MWh)	Charge HEXs (MWh)	Pumps (MWh)	Throttle (MWh)	Turbines (MWh)	Discharge HEXs (MWh)	Total (MWh)
1	380.42	450.09	10.50	116.85	408.28	233.86	1600.00
2	121.28	79.57	4.13	20.32	106.73	40.03	372.06
3	160.70	108.91	5.35	28.99	143.26	56.38	503.60
4	101.59	60.44	3.58	15.37	87.14	30.90	299.02
5	355.26	395.34	9.98	105.83	372.38	210.16	1448.94
6	316.61	318.23	9.24	85.66	320.41	166.08	1216.23
7	141.70	116.41	4.45	30.74	134.86	58.71	486.86
8	325.04	384.56	8.98	101.95	353.57	205.50	1379.59
9	218.77	226.83	6.31	59.65	223.74	115.36	850.66
10	348.55	432.63	9.40	112.73	382.03	232.44	1517.78

**Table 6:** System 3 charge phase thermodynamic states.

State	Pressure (kPa)	Temperature (K)	Enthalpy (kJ/kg)	Mass Flow Rate (kg/s)	Specific Exergy (kJ/kg)
1	101.32	293.15	294.95	108.00	0.00
2	415.41	454.88	465.68	108.00	152.13
3	398.80	301.24	304.57	108.00	115.32
4	1635.06	467.42	482.59	108.00	272.13
5	1569.66	301.86	310.74	108.00	230.29
6	6435.60	468.40	495.28	108.00	388.13
7	6178.18	301.91	332.74	108.00	344.55
8	101.32	293.15	0.01	89.89	0.00
9	101.32	293.15	0.01	89.89	0.00
10	101.32	293.15	0.01	179.79	0.00
11	182.38	293.40	0.46	179.79	0.08
12	182.38	293.40	0.46	62.04	0.08
13	182.38	293.40	0.46	60.70	0.08
14	182.38	293.40	0.46	57.05	0.08
15	175.08	446.80	280.93	62.04	57.43
16	175.08	458.72	306.20	60.70	66.34
17	175.08	459.65	308.17	57.05	67.05
18	175.08	454.90	298.11	179.79	63.41
19	175.08	454.90	298.11	89.89	63.41
20	175.08	454.90	298.11	89.89	63.41

**Table 7:** System 7 charge phase thermodynamic states.

State	Pressure (kPa)	Temperature (K)	Enthalpy (kJ/kg)	Mass Flow Rate (kg/s)	Specific Exergy (kJ/kg)
1	101.32	293.15	294.95	108.00	0.00
2	455.94	468.01	480.21	108.00	164.88
3	437.70	301.89	305.42	108.00	123.16
4	1969.66	481.97	499.37	108.00	293.57
5	1890.87	302.59	312.98	108.00	245.90
6	8508.93	483.09	515.44	108.00	417.91
7	8168.58	302.65	342.55	108.00	367.77
8	101.32	293.15	0.01	88.35	0.00
9	101.32	293.15	0.01	88.35	0.00
10	101.32	293.15	0.01	176.71	0.00
11	182.38	293.40	0.46	176.71	0.08
12	182.38	293.40	0.46	61.50	0.08
13	182.38	293.40	0.46	59.97	0.08
14	182.38	293.40	0.46	55.24	0.08
15	175.08	459.28	307.40	61.50	66.77
16	175.08	472.54	336.15	59.97	77.43
17	175.08	473.60	338.47	55.24	78.31
18	175.08	468.26	326.87	176.71	73.90
19	175.08	468.26	326.87	88.35	73.90
20	175.08	468.26	326.87	88.35	73.90

**Table 8:** System 3 discharge phase thermodynamic states.

State T = 0	Pressure (kPa)	Temp (K)	Enthalpy (kJ/kg)	Mass Flow Rate (kg/s)	Specific Exergy (kJ/kg)	State T = end	Pressure (kPa)	Temp (K)	Enthalpy (kJ/kg)	Mass Flow Rate (kg/s)	Specific Exergy (kJ/kg)
1	6094.52	346.10	371.99	108.00	347.91	1	3732.66	305.00	323.97	108.00	302.76
2	3732.66	354.52	371.99	108.00	308.45	2	3732.66	305.00	323.97	108.00	302.76
3	3583.36	462.26	481.88	108.00	336.25	3	3583.36	459.78	479.23	108.00	335.31
4	731.30	310.42	315.38	108.00	166.63	4	731.30	308.76	313.71	108.00	166.54
5	702.05	460.05	472.11	108.00	198.19	5	702.05	459.97	472.02	108.00	198.16
6	143.27	308.94	311.21	108.00	29.55	6	143.27	308.89	311.15	108.00	29.55
7	101.32	467.93	326.02	57.22	73.56	7	101.32	467.93	326.02	58.00	73.56
8	101.32	467.93	326.02	57.22	73.56	8	101.32	467.93	326.02	58.00	73.56
9	101.32	467.93	326.02	114.44	73.56	9	101.32	467.93	326.02	116.01	73.56
10	182.38	468.32	326.93	114.44	73.96	10	182.38	468.32	326.93	116.01	73.96
11	182.38	468.32	326.93	55.35	73.96	11	182.38	468.32	326.93	56.86	73.96
12	182.38	468.32	326.93	59.09	73.96	12	182.38	468.32	326.93	59.15	73.96
13	175.08	360.21	112.52	55.35	11.50	13	175.08	313.17	32.03	56.86	1.12
14	175.08	318.32	40.47	59.09	1.73	14	175.08	316.74	37.87	59.15	1.53
15	175.08	338.58	75.32	114.44	5.39	15	175.08	314.27	35.00	116.01	1.24
16	175.08	338.58	75.32	57.22	5.39	16	175.08	314.27	35.00	58.00	1.24
17	175.08	338.58	75.32	57.22	5.39	17	175.08	314.27	35.00	58.00	1.24

**Table 9:** System 7 discharge phase thermodynamic states.

State T = 0	Pressure (kPa)	Temp (K)	Enthalpy (kJ/kg)	Mass Flow Rate (kg/s)	Specific Exergy (kJ/kg)	State T = end	Pressure (kPa)	Temp (K)	Enthalpy (kJ/kg)	Mass Flow Rate (kg/s)	Specific Exergy (kJ/kg)
1	7905.10	347.37	379.70	108.00	369.82	1	4840.24	306.34	330.36	108.00	324.44
2	4840.24	357.88	379.70	108.00	330.78	2	4840.24	306.34	330.36	108.00	324.44
3	4646.63	466.64	489.19	108.00	359.87	3	4646.63	464.06	486.43	108.00	358.87
4	829.76	303.38	308.76	108.00	176.91	4	829.76	301.71	307.08	108.00	176.85
5	796.56	463.92	308.76	108.00	210.27	5	796.56	463.83	476.50	108.00	210.23
6	142.24	301.61	308.76	108.00	28.66	6	142.24	301.56	303.69	108.00	28.65
7	101.32	472.37	335.71	56.67	77.21	7	101.32	472.37	335.71	57.46	77.21
8	101.32	472.37	335.71	56.67	77.21	8	101.32	472.37	335.71	57.46	77.21
9	101.32	472.37	335.71	113.34	77.21	9	101.32	472.37	335.71	114.93	77.21
10	182.38	472.76	336.63	113.34	77.62	10	182.38	472.76	336.63	114.93	77.62
11	182.38	472.76	336.63	54.25	77.62	11	182.38	472.76	336.63	55.78	77.62
12	182.38	472.76	336.63	59.09	77.62	12	182.38	472.76	336.63	59.15	77.62
13	175.08	363.62	118.67	54.25	12.66	13	175.08	314.66	34.45	55.78	1.29
14	175.08	311.85	29.88	59.09	0.99	14	175.08	310.26	27.29	59.15	0.84
15	175.08	336.63	72.38	113.34	4.95	15	175.08	311.35	30.76	114.93	0.94
16	175.08	336.63	72.38	56.67	4.95	16	175.08	311.35	30.76	57.46	0.94
17	175.08	336.63	72.38	56.67	4.95	17	175.08	311.35	30.76	57.46	0.94

## Abbreviations

The following abbreviations are used in this manuscript:

A-CAES	Adiabatic-CAES
BGS	British Geological Survey
D-CAES	Diabatic-CAES
CAES	Compressed Air Energy Storage
CHT	Convective Heat Transfer
ES	Energy Storage
GIS	Graphical Information System
HEX	Heat Exchanger
I-CAES	Isothermal CAES
MOL	Method of Lines
PBTES	Packed Bed Thermal Energy Storage
RTE	Round Trip Efficiency
SC-CAES	Super Critical-CAES
TES	Thermal Energy Storage

## References

[1] Geissbühler L., Becattini V., Zanganeh G., Zavattoni S., Barbato M., Haselbacher A., Steinfeld A. (2018). Pilot-scale demonstration of advanced adiabatic compressed air energy storage, Part 1: Plant description and tests with sensible thermal-energy storage. J. Energy Storage.

[2] Wang S., Zhang X., Yang L., Zhou Y., Wang J. (2016). Experimental study of compressed air energy storage system with thermal energy storage. Energy.

[3] Kim Y.M., Shin D.G., Favrat D. (2011). Operating characteristics of constant-pressure compressed air energy storage (CAES) system combined with pumped hydro storage based on energy and exergy analysis. Energy.

[4] Guanwei J., Weiqing X., Maolin C., Yan S. (2018). Micron-sized water spray-cooled quasi-isothermal compression for compressed air energy storage. Exp. Therm. Fluid Sci..

[5] Heidari M., Mortazavi M., Rufer A. (2017). Design, modeling and experimental validation of a novel finned reciprocating compressor for Isothermal Compressed Air Energy Storage applications. Energy.

[6] Guo H., Xu Y., Chen H., Zhou X. (2016). Thermodynamic characteristics of a novel supercritical compressed air energy storage system. Energy Conv. Manag..

[7] Guo H., Xu Y., Chen H., Guo C., Qin W. (2017). Thermodynamic analytical solution and exergy analysis for supercritical compressed air energy storage system. Appl. Energy.

[8] Cheung B.C., Carriveau R., Ting D.S. (2014). Parameters affecting scalable underwater compressed air energy storage. Appl. Energy.

[9] Pimm A., Garvey S., de Jong M. (2014). Commercial grid scaling of Energy Bags for underwater compressed air energy storage. Int. J. Environ. Stud..

[10] Luo X., Wang J., Dooner M., Clarke J., Krupke C. (2014). Overview of current development in compressed air energy storage technology. Energy Procedia.

[11] Chen H., Cong T.N., Yang W., Tan C., Li Y., Ding Y. (2009). Progress in electrical energy storage system: A critical review. Pro. Nat. Sci..

[12] Sabihuddin S., Kiprakis A.E., Mueller M. (2015). A numerical and graphical review of energy storage technologies. Energies.

[13] Venkataramani G., Parankusam P., Ramalingam V., Wang J. (2016). A review on compressed air energy storage—A pathway for smart grid and polygeneration. Renew. Sustain. Energy Rev..

[14] Guney M.S., Tepe Y. (2017). Classification and assessment of energy storage systems. Renew. Sustain. Energy Rev..

[15] Aneke M., Wang M. (2016). Energy storage technologies and real life applications—A state of the art review. Appl. Energy.

[16] Amirante R., Cassone E., Distaso E., Tamburrano P. (2017). Overview on recent developments in energy storage: Mechanical, electrochemical and hydrogen technologies. Energy Conv. Manag..

[17] Luo X., Wang J., Dooner M., Clarke J. (2015). Overview of current development in electrical energy storage technologies and the application potential in power system operation. Appl. Energy.

[18] Mahlia T.M., Saktisahdan T.J., Jannifar A., Hasan M.H., Matseelar H.S. (2014). A review of available methods and development on energy storage; Technology update. Renew. Sustain. Energy Rev..

[19] Akinyele D.O., Rayudu R.K. (2014). Review of energy storage technologies for sustainable power networks. Sustain. Energy Techn..

[20] Zakeri B., Syri S. (2015). Electrical energy storage systems: A comparative life cycle cost analysis. Renew. Sustain. Energy Rev..

[21] Raju M., Kumar Khaitan S. (2012). Modeling and simulation of compressed air storage in caverns: A case study of the Huntorf plant. Appl. Energy.

[22] Kim Y.M., Lee J.H., Kim S.J., Favrat D. (2012). Potential and evolution of compressed air energy storage: Energy and exergy analyses. Entropy.

[23] Szablowski L., Krawczyk P., Badyda K., Karellas S., Kakaras E., Bujalski W. (2017). Energy and exergy analysis of adiabatic compressed air energy storage system. Energy.

[24] Liu J.L., Wang J.H. (2016). A comparative research of two adiabatic compressed air energy storage systems. Energy Conv. Manag..

[25] Luo X., Wang J., Krupke C., Wang Y., Sheng Y., Li J., Xu Y., Wang D., Miao S., Chen H. (2016). Modelling study, efficiency analysis and optimisation of large-scale Adiabatic Compressed Air Energy Storage systems with low-temperature thermal storage. Appl. Energy.

[26] Yang K., Zhang Y., Li X., Xu J. (2014). Theoretical evaluation on the impact of heat exchanger in Advanced Adiabatic Compressed Air Energy Storage system. Energy Conv. Manag..

[27] Peng H., Yang Y., Li R., Ling X. (2016). Thermodynamic analysis of an improved adiabatic compressed air energy storage system. Appl. Energy.

[28] Barbour E., Mignard D., Ding Y., Li Y. (2015). Adiabatic Compressed Air Energy Storage with packed bed thermal energy storage. Appl. Energy.

[29] He W., Wang J., Wang Y., Ding Y., Chen H., Wu Y., Garvey S. (2017). Study of cycle-to-cycle dynamic characteristics of adiabatic Compressed Air Energy Storage using packed bed Thermal Energy Storage. Energy.

[30] Mazloum Y., Sayah H., Nemer M. (2017). Exergy analysis and exergoeconomic optimization of a constant-pressure adiabatic compressed air energy storage system. J. Energy Storage.

[31] Chen L.X., Xie M.N., Zhao P.P., Wang F.X., Hu P., Wang D.X. (2018). A novel isobaric adiabatic compressed air energy storage (IA-CAES) system on the base of volatile fluid. Appl. Energy.

[32] Zhao P., Gao L., Wang J., Dai Y. (2016). Energy efficiency analysis and off-design analysis of two different discharge modes for compressed air energy storage system using axial turbines. Renew. Energy.

[33] Wang Z., Xiong W., Ting D.S., Carriveau R., Wang Z. (2016). Conventional and advanced exergy analyses of an underwater compressed air energy storage system. Appl. Energy.

[34] Mohammadi A., Ahmadi M.H., Bidi M., Joda F., Valero A., Uson S. (2017). Exergy analysis of a Combined Cooling, Heating and Power system integrated with wind turbine and compressed air energy storage system. Energy Conv. Manag..

[35] Parkes D., Evans D.J., Williamson P., Williams J.D.O. (2018). Estimating available salt volume for potential CAES development: A case study using the Northwich Halite of the Cheshire Basin. J. Energy Storage.

[36] He W., Luo X., Evans D., Busby J., Garvey S., Parkes D., Wang J. (2017). Exergy storage of compressed air in cavern and cavern volume estimation of the large-scale compressed air energy storage system. Appl. Energy.

[37] Dincer I., Rosen M. (2007). Exergy: Energy, Environment and Sustainable Development.

